# Less screen time and more frequent vigorous physical activity is associated with lower risk of reporting negative mental health symptoms among Icelandic adolescents

**DOI:** 10.1371/journal.pone.0196286

**Published:** 2018-04-26

**Authors:** Soffia M. Hrafnkelsdottir, Robert J. Brychta, Vaka Rognvaldsdottir, Sunna Gestsdottir, Kong Y. Chen, Erlingur Johannsson, Sigridur L. Guðmundsdottir, Sigurbjorn A. Arngrimsson

**Affiliations:** 1 Center of Sport and Health Sciences, University of Iceland, Reykjavik, Iceland; 2 Diabetes, Endocrinology and Obesity Branch, National Institute of Diabetes and Digestive and Kidney Diseases, Bethesda, MD, United States of America; 3 Department of Sport and Physical Activity, Bergen University College, Bergen, Norway; Maastricht University, NETHERLANDS

## Abstract

**Objective:**

Few studies have explored the potential interrelated associations of screen time and physical activity with mental health in youth, particularly using objective methods. We examined cross-sectional associations of these variables among Icelandic adolescents, using objective and subjective measurements of physical activity.

**Methods:**

Data were collected in the spring of 2015 from 315 tenth grade students (mean age 15.8 years) in six elementary schools in metropolitan Reykjavík, Iceland. Participants reported, via questionnaire, on demographics, weekly frequency of vigorous physical activity, daily hours of screen time and mental health status (symptoms of depression, anxiety and somatic complaints, self-esteem and life satisfaction). Total physical activity was measured over one week with wrist-worn accelerometers. Body composition was determined by DXA-scanning. Poisson regression analysis was used to explore independent and interactive associations of screen time and physical activity with mental health variables, adjusting for gender, body fat percentage and maternal education.

**Results:**

Less screen time (below the group median of 5.3 h/day) and more frequent vigorous physical activity (≥4x/week) were each associated with reporting fewer symptoms of depression, anxiety, low self-esteem, and life dissatisfaction. No significant associations were observed between objectively measured physical activity and mental health outcomes. Interactive regression analysis showed that the group reporting both less screen time and more frequent vigorous physical activity had the lowest risk of reporting symptoms of depression, anxiety, low self-esteem, and life dissatisfaction.

**Conclusions:**

Reports of less screen time and more frequent vigorous physical activity were associated with lower risk of reporting mental health problems among Icelandic adolescents. Those who reported a combination of engaging in less screen time and more frequent vigorous physical activity had the lowest risk, suggesting a synergistic relationship between the two behaviors on mental health outcomes. Our results support guiding youth towards more active and less sedentary/screen-based lifestyle.

## Introduction

Mental health may be positively influenced by self-esteem and life satisfaction but negatively affected by mental disorders, such as depression, anxiety and somatic complaints [1]. The prevalence of mental disorders, including depression and anxiety, has increased over the past decades [2] and now accounts for 30% of the global non-fatal disease burden, affecting close to 10% of the world´s population [3]. Prevalence of mental health problems rises sharply in adolescence [4,5], with depression and anxiety being among the leading causes of the burden of disease and disability in youth [6]. These syndromes often manifest physically as somatic complaints [7]. A study on the secular trend in symptoms of depression and anxiety among Icelandic adolescents reported increases in symptoms and more frequent visits to psychiatrists, psychologists and social workers during the period 1997–2006 [8]. Poor life satisfaction is strongly associated with mortality in adults [9] and psychosocial and behavioral problems [10] and violence [11] in adolescents. Mental well-being is highly affected by self-esteem among adolescents [12] and low self-esteem has been linked to increased depression and anxiety [13]. Depression and anxiety are strong predictors of negative health and psychosocial outcomes, including academic difficulties, behavioral problems, low self-esteem, substance abuse, and suicide [14]. Furthermore, youth experiencing anxiety and depression are at significantly increased risk of these conditions in adulthood [14]. The identification of risk factors and/or protective factors during adolescence is therefore a very important public health issue.

Parallel to the rise in mental health disorders in the past decades has been an increased usage of leisure-time screen based media [15] and a considerable reduction in the level of physical activity [16,17], particularly in adolescents [17–19]. Not surprisingly, excessive screen-based activities and limited physical activity have been proposed as potential risk factors for mental health problems [20–22]. Studies examining the effects of both screen time and physical activity on mental health have typically investigated their separate effects but not their potential interrelated effects [20,23]. These factors may operate synergistically to affect mental health, high levels of screen time and low levels of physical activity have been found to interact and lead to increased mental health problems among college students [24,25] and in children during pre- or early adolescence [26–28]. Less is known about these interactive effects in youngsters in their mid-teens. Further, most prior studies have relied solely on self-reported physical activity, rather than using objective measures.

The purpose of this study was to examine both the separate and interactive associations of screen time and physical activity with self-reported mental health in Icelandic adolescents. We hypothesized that: 1) study participants with fewer hours of screen time would be less likely to report experiencing symptoms of poor mental health, 2) participants with higher levels of physical activity would also be less likely to report having symptoms of poor mental health, and 3) participants with both fewer hours of screen time and higher levels of physical activity would have the lowest risk of reporting mental health problems.

## Methods

### Sample and data collection

Four hundred and eleven tenth-grade students (age 15–16 years, 47% boys and 53% girls) from six elementary schools in metropolitan Reykjavik, Iceland, were invited to participate in the study; 315 (79%) of which agreed to participate. Non-participation was mainly due to absence from school during measurement days and lack of interest in the study. Data collection was performed between mid-April and early June of 2015. Participants provided information regarding their background, health and lifestyle by answering a tablet-based questionnaire (in Icelandic) administered at school under the supervision of research team members. The questionnaire addressed age, sex, maternal education (as a proxy for socioeconomic status), participation in screen-based activities, weekly frequency of vigorous physical activity, symptoms of mental health problems (depression, anxiety and somatic complaints), self-esteem and life satisfaction. Objective measurements of free-living physical activity, weight, height and body composition were also performed. Written informed consent was obtained from all participants and their guardians. Strict procedures were followed to ensure confidentiality. The research project was approved by the Icelandic Data Protection Authority and the National Bioethics Committee as well as the Icelandic Radiation Safety Authority.

### Exposure measures

#### Self-reported vigorous physical activity

Participants were asked the following question: „How often, per week, do you perform physical activity that makes you breathe more rapidly or sweat? The variable was scored on a six-point Likert scale, with the following response options: 1 = “never”, 2 = “less than once a week”, 3 = “once a week”, 4 = “2–3 times a week”, 5 = “4–5 times a week”, 6 = “almost every day”. For analysis, the variable was recoded using the following two categories: Less frequently = “less than 4 times a week” and more frequently = “4 times a week or more”, based on international physical activity guidelines stating that children and adolescents should participate in vigorous-intensity physical activity at least 3 days a week [29].

#### Objectively measured physical activity

Free-living physical activity was objectively measured using small (3.8 cm x 3.7 cm x 1.8 cm) and lightweight (27 g) triaxial raw signal accelerometer-based Actigraph activity monitors (model GT3X+ ActiSleep, Actigraph Inc. Pensacola Florida). Each participant was asked to continuously wear the monitor on his/her non-dominant wrist for 7 consecutive days. A minimum of 3 valid schooldays and 1 valid non-schoolday was set as an inclusion criterion. Days with a wear-time of ≥ 14 h from 12 midnight to 12 midnight the following day were considered valid. Raw triaxial data (in milliG‘s) sampled at 80 samples per second (Hz) were reduced to the vector magnitude of activity counts over 60 s epoch and averaged over all valid days using Actilife software from Actigraph (Pensacola, FL, USA; version 6.13.0) and customized programs in Matlab (The Mathworks, Natick, MA, USA; version R2013a). Participants were categorized as having higher and lower levels of objective total physical activity, using the group median value as a cut-off.

#### Self-reported screen time

Participants were asked to report on how many hours per day on average, separately for weekdays and weekend-days, they played computer games, watched TV/DVD/internet material, used the internet for web-browsing/Facebook/e-mail and participated in „other”computer use. Each item was scored on a seven-point Likert scale, with the following response options: 1 = “none”, 2 = “about ½ h”, 3 = “1 up to 2 h”, 4 = “2 up to 3 h”, 5 = “3 up to 4 h”, 6 = “4 to 5 h” and 7 = “more than 5 h”. Average daily hours for each type of screen-based activity were computed, using the midpoints for scoring categories and weighted averages for weekdays and weekend-days. All screen-based activities were then summed for a total daily screen time (h/day) and participants were sorted into high and low screen time groups based on their relation to the group median value.

### Outcome measures

#### Mental health problems

A 22-item version of the Subscales of the Symptom Checklist 90 (SCL-90) [30] was used to assess symptoms of depression (10 items), anxiety (4 items) and somatic complaints (8 items). Participants were asked how often they had experienced symptoms of these conditions during the preceding week. Each item was rated on a five-point Likert scale: 1 = “almost never“, 2 = “seldom“, 3 = “sometimes“, 4 = “often”and 5 = “almost always“. The following cut-offs, based on the midpoint of possible scores, were used to define a healthy versus an unhealthy score: depression symptoms ≥ 30 points, anxiety symptoms ≥ 12 points and somatic complaints ≥ 24 points. This 22-item version of the SCL-90, using the same cut-offs, has previously been employed in a study on mental well-being among Icelandic adolescents [31].

#### Global self-esteem

Global self-esteem was assessed using the Rosenberg Self-Esteem Scale [32]. The scale consists of 10 statements, each rated as positive or negative, with four response options: 0 = “strongly agree“, 1 = “somewhat agree“, 2 = “somewhat disagree”and 3 = “strongly disagree“. A score ≥ 15 points reflects a greater level of self-esteem. The Rosenberg scale has been widely used for evaluating self-esteem of young people, and its reliability and validity are well documented [33].

#### Life satisfaction

The Diener´s Satisfaction with Life Scale (SWLS), a measure of global cognitive judgments of satisfaction with one's life, was used for estimating life satisfaction of participants [34]. The scale contains 5 items rated on a 7-point Likert scale, with the following response options: 1 = “strongly disagree“, 2 = “disagree”, 3 = “somewhat disagree“, 4 = “neither agree nor disagree“, 5 = “somewhat agree“, 6 = “agree“, 7 = “strongly agree“. A score of 20 represents a neutral point on the scale, with higher score indicating more satisfaction and lower score indicating less satisfaction. Scores on the SWLS have been shown to correlate with measures of mental health and the scale is reported to have a high internal consistency and good test-retest correlations [35].

### Covariates

Body composition and maternal education were selected as covariates, based on prior studies and our own correlation analysis. Previous research has identified associations between body composition and screen time [36–38], physical activity [38,39] and mental health [40–42]. Our data confirm these associations, as body composition was significantly correlated with physical activity, screen time, and all the mental health outcomes. Education of parents, especially mothers, has been found to associate with the mental health of their offspring [43]. Maternal education is an indicator of socioeconomic position, which may affect youngsters’ participation in recreational activities, including screen-based activities [44] and physical activity [45]. Furthermore, low educational attainment of parents has been shown to independently associate with less utilization of child mental health resources, and increased severity and duration of mental health problems of children [46,47].

#### Body composition

Body mass index (BMI, kg/m^2^) was calculated from measurements of weight (kg) and height (m). Standing height was measured to the nearest mm with a transportable stadiometer (Seca model 217, Seca Ltd., Birmingham, UK). Body weight was measured to the nearest 0.1 kg on a calibrated scale (Seca model 813, Seca Ltd., Birmingham, UK), with participants wearing light clothing. These measurements were performed at individual schools. Whole-body and regional soft tissue composition was measured by dual energy X-ray absorptiometry (DXA) using a Lunar bone densitometer (GE Healthcare, Madison, Wisconsin USA) to obtain percentage body fat. All DXA-scans were run by the same certified radiologist at the facilities of the Icelandic Heart Association in Kopavogur, Iceland.

#### Maternal education

Educational level of mother was reported by the participants, given the following seven categories to choose from: 1 = “elementary degree”, 2 = “secondary degree”, 3 = “trade school degree”, 4 = “university degree”, 5 = “other”, 6 = “do not know”, 7 = “do not want to answer”. These options were recoded into a new binary variable, university education of mother, with the following categories: 1 = “having university degree” and 0 = “not having university degree”.

### Statistical analysis

Descriptive summaries are presented as means and standard deviations for continuous variables and as frequencies and percentages for categorical variables. Sex differences were evaluated by t-tests for continuous variables and chi-square tests for categorical variables. Pearson‘s correlational analysis was used to evaluate relationships between the main variables of interest. Poisson regression analysis was performed to calculate the relative risk (RR) and 95% confidence intervals (CIs) of reporting symptoms of depression, anxiety, somatic complaints, low self-esteem, and life dissatisfaction with respect to screen time, frequency of self-reported vigorous physical activity, and objectively measured total physical activity. First, separate Poisson regressions were performed between each independent variable (i.e. total daily screen time, subjective vigorous physical activity, and objective total physical activity) and each mental health outcome. The sample was then divided into the following four groups, based on the total daily screen time and subjective vigorous physical activity, for interactive Poisson regression analysis: higher screen time + less frequent vigorous physical activity (reference group), higher screen time + more frequent vigorous physical activity, lower screen time + less frequent vigorous physical activity and lower screen time + more frequent vigorous physical activity. Since objective total physical activity was not associated with the mental health outcomes in the univariate analysis, an analogous interactive analysis using screen time and objective total physical activity was not performed. All Poisson regression models were adjusted for the following potential confounders: sex, body fat percentage and maternal education. Significant differences or relations were accepted at α < 0.05. Statistical analyses were performed using SAS statistical software, version 9.4 (SAS Institute Inc., Cary, NC; www.sas.com).

## Results

### Characteristics of participants

The inclusion criteria of valid measurements for free-living physical activity (a minimum of 3 weekdays and 1 weekend day) was fulfilled for 272 participants, 248 of which also had complete self-reported data for screen time, vigorous physical activity and mental health variables. Four participants had values for screen time exceeding the upper limit for that variable (mean + 2.5 SD) and were thus removed as outliers. The removal did not meaningfully change the results of our regression analyses. The final study sample consisted of 244 participants, 100 boys and 144 girls.

Characteristics of participants are shown in Table 1, for the total group as well as boys and girls separately. The mean age of participants was 15.8 years. Average total screen time was 5.8 ± 2.5 h/day (median 5.3 h/day), significantly greater for boys than girls (p = 0.04). Spending between 4 and 6 h/day in screen based activities was most common, for both sexes (around 40%), and only a very small minority reported screen time below 2 h/day. Almost two-thirds reported engaging in vigorous physical activity ≥ 4 times a week. Prevalence of symptoms of depression, anxiety, and somatic complaints was significantly higher among girls (15–22%) than boys (3–8%). Low self-esteem was reported by 16% and life dissatisfaction by 20% of participants. Average value for BMI was 21.9 kg/m^2^, the prevalence of being overweight (BMI between 25 and 30 kg/m^2^) was approximately 11% and around 2% of the group were obese (BMI ≥ 30 kg/m^2^). Average body fat percentage was 25.3%, significantly higher for girls than boys (p < 0.0001).

**Table 1:** Characteristics of the study subjects.

	Total (n = 244)	Males (n = 100)	Females (n = 144)	p-value^3^
Age, years, mean (SD)	15.8 (0.3)	15.8 (0.3)	15.9 (0.3)	0.07
BMI, kg/m^2^, mean (SD)	21.9 (3.1)	21.5 (2.8)	22.3 (3.2)	0.06
Normal (BMI: 20 < x ≤ 25), n (%)	193 (79.4)	83 (83.8)	110 (76.4)	
Overweight (BMI: 25 < x ≤ 30), n (%)	26 (10.7)	7 (7.1)	19 (13.2)	
Obese (BMI: 30 < x), n (%)	5 (2.1)	1 (1.0)	4 (2.8)	
Underweight (BMI: x ≤ 20), n (%)	19 (7.8)	8 (8.1)	11 (7.6)	
Body fat, %, mean (SD)	25.3 (9.0)	17.7 (6.6)	30.5 (6.3)	**< 0.0001**
Vigorous PA, subjective, n (%)				0.12
Less (< 4x/week)	87 (35.7)	30 (30.0)	57 (39.6)	
More (≥ 4x/week)	157 (64.3)	70 (70.0)	87 (60.4)	
Total PA^1^, objective, cpm/day, mean (SD)	2039 (472)	1993 (452)	2071 (484)	0.20
Below median (N = 122; 53 males, 69 females)	1673 (241)	1650 (244)	1691 (239)	0.34
Above median (N = 122; 47 males, 75 females)	2404 (345)	2380 (289)	2420 (378)	0.53
Screen time^2^, h/day, mean (SD)	5.8 (2.5)	6.2 (2.4)	5.5 (2.6)	**0.04**
Below median (N = 122; 42 males, 80 females)	3.8 (1.1)	4.1 (0.9)	3.7 (1.1)	0.12
Above median (N = 122; 58 males, 64 females)	7.7 (2.0)	7.7 (2.0)	7.7 (2.1)	0.74
Screen time categories, h/day, n (%)				0.24
x ≤ 2	8 (3.3)	1 (1.0)	7 (4.9)	
2 < x ≤ 4	49 (20.1)	16 (16.0)	33 (22.9)	
4 < x ≤ 6	99 (40.6)	42 (42.0)	57 (39.6)	
6 < x ≤ 8	45 (18.4)	20 (20.0)	25 (17.4)	
8 < x	43 (17.6)	21 (21.0)	22 (15.3)	
Depression				
Score, mean (SD)	17.6 (8.9)	14.4 (6.3)	19.7 (9.8)	**< 0.0001**
Symptoms (score > 30), n (%)	26 (10.7)	4 (4.0)	22 (15.3)	**< 0.005**
Anxiety				
Score, mean (SD)	6.6 (3.6)	5.3 (2.5)	7.6 (4.0)	**< 0.0001**
Symptoms (score > 12), n (%)	34 (13.9)	3 (3.0)	31 (21.5)	**< 0.0001**
Somatic complaints				
Score, mean (SD)	15.6 (6.6)	13.1 (5.2)	17.3 (7.0)	**< 0.0001**
Symptoms (score > 24), n (%)	35 (14.3)	8 (8.0)	27 (18.8)	**0.02**
Self-esteem				
Score, mean (SD)	21.3 (6.8)	22.1 (7.0)	20.7 (6.7)	0.11
Low self-esteem (score < 15), n (%)	38 (15.6)	12 (12.0)	26 (18.1)	0.20
Life satisfaction				
Score, mean (SD)	26.1 (7.1)	26.1 (7.7)	26.1 (6.6)	0.99
Life dissatisfaction (score ≤ 20), n (%)	48 (19.7)	24 (24.0)	24 (16.7)	0.16
Maternal education				
University education	146 (59.8)	66 (66.0)	80 (55.6)	0.10
No university education	98 (40.2)	34 (34.0)	64 (44.4)	

BMI = body mass index, PA = physical activity, SD = standard deviation

^1^mean of total vector magnitude activity/wear time; total group median = 2039 cpm/day

^2^weighted average for the week (5 weekdays and 2 weekend-days); total group median = 5.3 h/day

^3^p-value < 0.05, for differences between sexes, was considered statistically significant

### Regression analysis

Tables 2 and 3 show the associations of screen time, self-reported vigorous physical activity and objectively measured physical activity with self-reported measures of mental health status, i.e. symptoms of anxiety, depression, and somatic complaints (Table 2) and self-esteem and life dissatisfaction (Table 3). After adjustment for potential confounders (sex, maternal education and percentage body fat), reporting less screen time was associated with a significantly lower risk of reporting symptoms of depression (RR = 0.33, 95% CI = 0.14–0.76), anxiety (RR = 0.44, 95% CI = 0.23–0.84), low self-esteem (RR = 0.31, 95% CI = 0.15–0.66) and life dissatisfaction (RR = 0.38, 95% CI = 0.20–0.72). Self-reported vigorous physical activity showed similar associations (Tables 2 and 3), but objectively measured physical activity was not associated with any of the mental health outcomes (despite being positively correlated to self-reported vigorous physical activity (see S1 and S2 Tables). The relative risk of reporting somatic complaints was marginally lower for those reporting lower screen time (RR = 0.55, 95% CI = 0.29–1.03) but this outcome was unrelated to both self-reported vigorous physical activity and objectively measured physical activity (Table 2).

**Table 2:** Associations of screen time and physical activity with symptoms of depression, anxiety and somatic complaints.

	Depressive symptoms		Anxiety symptoms		Somatic complaints	
	Yes, N (%^a^)	RR^b^ (95% CI)	Yes, N (%^a^)	RR^b^ (95% CI)	Yes, N (%^a^)	RR^b^ (95% CI)
**Total screen time**^c^						
Below median	7 (5.7)	0.33 (0.14, 0.76)***	12 (9.8)	0.44 (0.23, 0.84)**	13 (10.7)	0.55 (0.29, 1.03)
Above median	19 (15.6)	Ref.	22 (18.0)	Ref.	22 (18.0)	Ref.
**Vigorous PA**, subjective						
Less (< 4x/week)	17 (19.5)	Ref.	22 (25.3)	Ref.	14 (16.1)	Ref.
More (≥ 4x/week)	9 (5.7)	**0.31 (0.14, 0.71)** ***	12 (7.6)	**0.35 (0.18, 0.67)** ****	21 (13.4)	0.93 (0.50, 1.76)
**Total PA**, objective^d^						
Below median	11 (9.0)	Ref.	14 (11.5)	Ref.	17 (13.9)	Ref.
sAbove median	15 (12.3)	1.24 (0.60, 2.56)	20 (16.4)	1.38 (0.74, 2.54)	18 (14.8)	0.99 (0.53, 1.85)

RR = relative risk, CI = confidence interval, PA = physical activity

^a^Percent of the subgroup

^b^Adjusted for sex, body fat percentage and maternal education

^c^Median value for total screen time = 5.3 h/day

^d^Median value for total PA = 1975 cpm/day

**p < 0.05

***p < 0.01

****p <0.001

**Table 3:** Associations of screen time and physical activity with low self-esteem and life dissatisfaction.

	Low self-esteem		Life dissatisfaction	
	Yes, N (%^a^)	RR^b^ (95% CI)	Yes, N (%^a^)	RR^b^ (95% CI)
**Total screen time**^c^				
Below median	9 (7.4)	**0.31 (0.15, 0.66)** ^***^	12 (9.8)	0.38 (0.20, 0.72)^***^
Above median	29 (23.8)	Ref.	36 (29.5)	Ref.
**Vigorous PA**, subjective				
Less (<4x/week)	21 (24.1)	Ref.	25 (28.7)	Ref.
More (≥4x/week)	17 (10.8)	**0.48 (0.26, 0.90)** ^**^	23 (14.7)	**0.58 (0.35, 0.96)** ^**^
**Total PA**, objective^d^				
Below median	23 (18.9)	Ref.	27 (22.1)	Ref.
Above median	15 (12.3)	0.64 (0.35, 1.19)	21 (17.2)	0.84 (0.51, 1.39)

RR = relative risk, CI = confidence interval, PA = physical activity

^a^Percent of the subgroup

^b^Adjusted for sex, body fat percentage and maternal education

^c^Median value for total screen time = 5.3 h/day

^d^Median value for total PA = 1975 cpm/day

**p < 0.05

***p < 0.005

Dividing the participants into four subgroups based on both self-reported screen time and vigorous physical activity (Table 4) revealed that those reporting both less screen time and more frequent vigorous physical activity had a significantly lower risk of reporting symptoms of depression (RR = 0.06, 95% CI = 0.01–0.41), anxiety (RR = 0.16, 95% CI = 0.06–0.45), low self-esteem (RR = 0.16, 95% CI = 0.05–0.48) and life dissatisfaction (RR = 0.30, 95% CI = 0.15–0.61) compared with those reporting both greater screen time and less frequent vigorous physical activity, after adjusting for sex, maternal education and percentage body fat. A significantly lower risk of reporting life dissatisfaction was also found for those reporting having both less screen time and less frequent vigorous physical activity compared with those reporting more screen time and less frequent vigorous physical activity. No associations were observed between the combined screen time-vigorous physical activity subgroups and their reported somatic complaints.

**Table 4:** Interactive effects of screen time and vigorous PA on mental health.

VPA (x/wk)	Screen time (h/day)	N (subgroup)	Depressive Symptoms		Anxiety Symptoms		Somatic Complaints		Life dissatisfaction		Low Self-Esteem	
			Yes,N(%^a^)	RR^b^ (95% CI)	Yes, N(%^a^)	RR^b^ (95% CI)	Yes, N(%^a^)	RR^b^ (95% CI)	Yes, N(%^a^)	RR^b^ (95% CI)	Yes, N (%^a^)	RR^b^ (95% CI)
<4	> 5.3	54	11 (20.4)	Ref.	14 (25.9)	Ref.	8 (14.8)	Ref.	22 (40.7)	Ref.	16 (29.6)	Ref.
<4	< 5.3	33	6 (18.2)	0.87 (0.38, 2.00)	8 (24.2)	0.89 (0.44, 1.80)	6 (18.2)	1.25 (0.49, 3.19)	3 (9.1)	0.26 (0.08, 0.80)*	5 (15.2)	0.53 (0.21, 1.30)
≥4	> 5.3	68	8 (11.8)	0.74 (0.30, 1.84)	8 (11.8)	0.65 (0.29, 1.44)	14 (20.6)	1.78 (0.79, 4.01)	14 (20.6)	0.55 (0.30, 1.00)	13 (19.1)	0.73 (0.37, 1.45)
≥4	< 5.3	89	1 (1.1)	0.06 (0.01, 0.41)**	4 (4.5)	0.16 (0.06, 0.45)***	7 (7.9)	0.57 (0.22, 1.44)	9 (10.1)	0.30 (0.15, 0.61)**	4 (4.5)	0.16 (0.05, 0.48)**

PA = physical activity, VPA = vigorous physical activity, RR = relative risk, CI = confidence interval

^a^Percent of the subgroup

^b^Adjusted for sex, body fat percentage and maternal education

*p<0.05

**p<0.005

***p<0.0005

## Discussion

In this study, we observed that reports of less screen time and more frequent vigorous physical activity were each associated with a lower risk of reporting symptoms of depression, anxiety, low self-esteem, and life dissatisfaction. Furthermore, those who reported a combination of engaging in less screen time and more frequent vigorous physical activity had the lowest risk of reporting negative mental health symptoms, suggesting a synergistic relationship between the two behaviors on mental health outcomes.

We found that those who reported engaging in less than the median screen time of 5.3 h/day had a reduced risk of reporting symptoms of negative mental health compared with those reporting screen time greater than 5.3 h/day. This finding was relatively consistent across mental health outcomes, with reductions in relative risk ranging from 56–69% for reports of symptoms of depression and anxiety, low self-esteem, and life dissatistifaction. These results are in line with recent reviews reporting that increased participation in screen based activities in leisure time may be linked to poorer mental health among adolescents, including depressive symptomatology and psychological distress, decreased perceptions of self-worth, and lower perceived quality of life and self-esteem [21,22,37]. Our results support most of the previous findings in other adolescent age groups, of studies with similar design [23–25]. In a study by Trinh et al. [23] on Canadian youth (age 13–18 years, mean age 15.8 years), higher screen time was associated with symptoms of psychological distress (including anxiety) and depression, and lower self-esteem. Similarly, studies on Chinese college students found screen time to negatively impact reported symptoms of depression [24,25] and anxiety [25]. Consistent with our findings, a negative linear relationship between screen time and quality of life has been reported [48]. A study on Iranian youth [49] found, however, no association between screen time and life satisfaction. These prior studies define high screen time as greater than 2 h/day according to international recommendations. In the current study, however, the group median for total daily screen time was used as the cut-point since very few participants (n = 8 or 3.3%) averaged ≤ 2 h/day.

Participating in vigorous physical activity at least 4 times/week was associated with reduced risk (42–69%) of reporting the various mental health problems examined in our study (excluding somatic complaints). Previous findings on the impact of vigorous physical activity on mental health in youth have been mixed, perhaps due to variations in participant age and methodologies to evaluate physical activity and mental health outcomes [50]. In a study on Chinese college students [25], depression and anxiety were not found to be associated with frequency of physical activity alone, but were negatively associated with a measure that also accounted for intensity and duration of the activity. Further, self-reported vigorous or moderate-to-vigorous physical activity in adolescents was not found to be associated with symptoms of depression and anxiety [23,24] or low self-esteem [23]. Life satisfaction was, however, associated with self-reported physical activity in Iranian youth [49], and quality of life was related to self-reported weekly frequency of moderate-to-vigorous physical activity in the study by Iannotti et al. [48].

Those who reported a combination of less screen time and more frequent vigorous physical activity had the lowest risk of reporting negative mental health symptoms. This finding, for adolescents in their mid-teens, agrees with the results of the few prior studies on younger [26–28] and older adolescents [24,25]. The risk reduction for the less screen time-more frequent vigorous physical activity group, compared with the more screen time-less frequent vigorous physical activity group, was very substantial for symptoms of depression and anxiety (84–94%) and greater than that observed in the studies mentioned above for other age-groups of adolescents. The less screen time-more frequent vigorous physical activity group was also least likely to report low self-esteem (84% reduced risk), which supports prior findings by Trinh et al. [23]. Significantly lower risk of reporting life dissatisfaction was found in our study for those with less screen time, independent of the frequency of vigorous physical activity. In comparison, an interactive analysis conducted by Matin et al. [49] showed that the joint effect of low screen time and high physical activity was most strongly associated with life satisfaction in Iranian youth.

We observed a positive correlation between self-reported vigorous physical activity and objectively measured total activity (see S1 and S2 Tables). Despite this correlation, the objectively measured physical activity was surprisingly not associated with the mental health outcomes in our study. It is possible that the intensity of the activity must be above certain threshold to provide mental health benefits, although prior research has been inconclusive in this regard [51,52]. A recent review reported indications of beneficial effects of objectively measured total physical activity on mental health among adolescents [50]. However, it was concluded that relationships were more consistent and robust for higher versus lower intensity physical activity. Using self-reported physical activity, Mekary et al. [53] found that substituting 60 minutes/day of brisk/very brisk walking for television watching was more protective against depression than the same amount of average-paced walking. Their results also indicated that walking at an easy pace was not protective against depression. Conversely, in a review of exercise interventions to reduce or prevent anxiety or depression in youth, Larun et al. [54] concluded that exercise intensity had little impact on depression and anxiety scores in the general population of children and adolescents. Unfortunately, we were unable to obtain objective measures of vigorous activity since there are currently no agreed upon cut-points for wrist-worn accelerometers for youth.

It is a very important public health issue to prevent mental health problems in adolescents. As previously mentioned, depression and anxiety are not only strong predictors of negative health and psychosocial outcomes in adolescence but may also put individuals at a significantly increased risk of these conditions in adulthood [14]. Our findings suggest that limiting screen time and increasing participation in vigorous physical activity may separately, but especially in combination, have beneficial effects on mental health in adolescents. Although more detailed research is needed to confirm causality, these results support that official guidelines should not only include recommendations for promoting physical activity but also for limiting sedentary, screen based activities, to optimize mental health in youth.

Physical activity may have positive effects on mental health in adolescents via physiological, biochemical and psychological mechanisms [55]. It may improve mental health by having beneficial effects on body composition [40,42] and levels of mood-regulating neurotransmitters in the brain [56]. Regular physical activity may also promote mental health by improving self-esteem, self-efficacy and cognitive and psychological function, reducing distress [20,55], and increasing social interaction and support [55,57,58]. Increased screen time has been associated with poorer mental health among adolescents [21,22,37], potential mechanisms underlying such associations include: 1) negative effects of sedentary behavior on body composition [37]; 2) psychosocial and psychological effects, as media use via the internet provides adolescents with diverse opportunities for comparing themselves with others. Discrepancies between these publicized ideals and the self could cause social pressure and mental health problems [59]; 3) screen time may negatively affect sleep [60], which might have unfavorable effects on mental health [5]; 4) screen time may displace physical activity [61], resulting in loss of beneficial effects of exercise on mental health.

Our results contribute to the limited knowledge base of the interactive effects of screen time and physical activity on mental health among adolescents in their mid-teens, and this is the first study on this topic among Icelandic adolescents. A major strength of the study is the number and diversity of the mental health outcomes being evaluated; the agreement of results across these outcomes adds to the value of the study findings. Another strength of our study are the objective measurements of total physical activity by accelerometers, prior studies with a comparable study design have mostly evaluated physical activity by self-report alone. Still another strength is the use of DXA-measured body fat percentage as a covariate in our statistical analyses. The participation rate in the study (79%) was also quite high and the study sample represents a relatively large portion of the total number of 15 year old Icelandic adolescents in the year of 2015 (4,254 individuals, born in 1999) [62].

The cross-sectional study design does not allow us to determine causal relationships between the study variables. Reverse causality cannot be ruled out, participants reporting mental health problems might tend to be socially isolated and spend more time in screen based activities and less time in physical activity than their peers. Longitudinal studies are needed to further clarify causality between screen time, physical activity and mental health outcomes. Another limitation of the present study is the self-report of screen time and vigorous physical activity which is subject to recall and reporting biases. Our questionnaire included separate questions for time spent on individual screen based activities (games, TV/DVD watching, web/browsing/social-media/email, and other screen usage) which were combined for the total screen time used in our analyses. While this approach can potentially provide more detailed information on screen activities, this may have resulted in an over-estimation of total screen time, as multi-tasking on different screens, such as watching TV and using a smart-phone at the same time, is quite prevalent in youth [15]. It is also important to keep in mind that the questionnaire-based assessment of mental health used here is not equivalent to clinical diagnosis. Finally, although we have no evidence to suggest that the non-participants (N = 96) differed from the general student population, we cannot rule out the possibility of selection bias. Non-participants may have differed from participants in terms of socioeconomic status, lifestyle habits—including physical activity and screen time—and/or mental health status. But since they did not consent to the study we were unable to assess these potential differences.

In summary, we found that less screen time and more frequent vigorous physical activity are both separately and interactively associated with less risk of reporting symptoms of depression, anxiety, low self-esteem and life dissatisfaction in adolescents in their mid-teens. Our results support public health recommendations that guide youth towards more active and less sedentary/screen-based lifestyle. However, further research on causal relationships between physical activity, screen time and mental health is needed, i.e. longitudinal and intervention studies.

## Supporting information

S1 Table Correlation of screen time and self-reported vigorous PA with objective PA. (DOCX)

S2 Table Objective PA within subgroups based on reported screen time and vigorous PA. (DOCX)

S1 Dataset Raw data. (XLSX)
