# Supplementary material for: Less screen time and more frequent vigorous physical activity is associated with lower risk of reporting negative mental health symptoms among Icelandic adolescents
Source: PLoS One. 2018 Apr 26;13(4):e0196286. doi: 10.1371/journal.pone.0196286 (PMC5919516; doi:10.1371/journal.pone.0196286)
Supplement: S1 Table — (DOCX) [file pone.0196286.s001.docx]

**S1 table. Correlation of screen time and self-reported vigorous PA with objective PA**

| **Correlation** | **Pearson Correlation coefficient** | **p-value** |
| --- | --- | --- |
| Screen time vs. Objective PA | - 0.22 | 0.0005 |
| Vigorus PA vs. Objective PA | 0.27 | < 0.0001 |

PA = physical activity
