# Supplementary material for: Less screen time and more frequent vigorous physical activity is associated with lower risk of reporting negative mental health symptoms among Icelandic adolescents
Source: PLoS One. 2018 Apr 26;13(4):e0196286. doi: 10.1371/journal.pone.0196286 (PMC5919516; doi:10.1371/journal.pone.0196286)
Supplement: S2 Table — (DOCX) [file pone.0196286.s002.docx]

**S2 table. Objective PA within subgroups based on reported screen time and vigorous PA**

|  | **N (%)** | **PA objective, cpm/day, mean (SD)** | **p-value*** |
| --- | --- | --- | --- |
| **Screen time (ST)** |  |  |  |
| Lower half (<5.3 h/day) | 122 (50) | 2145 (482) | 0.0004 |
| Upper half (>5.3 h/day) | 122 (50) | 1933 (439) |  |
| **Vigorous physical activity (VPA)** |  |  | 0.0006 |
| More frequent (≥ 4x/week) | 157 (64.3) | 2115 (454) |  |
| Less frequent (< 4x/week) | 87 (35.7) | 1901 (475) |  |
| **Combined ST and VPA groups** |  |  | 0.0001 |
| Less ST – more frequent VPA | 89 (36.5) | 2189 (443)** |  |
| Less ST – less frequent VPA | 33 (13.5) | 2025 (562) |  |
| More ST – more frequent VPA | 68 (27.9) | 2019 (453) |  |
| More ST – less frequent VPA | 54 (22.1) | 1825 (399)** |  |

PA = physical activity, cpm = counts per minute, SD = standard deviation

*by t-test for comparison between individual ST and VPA groups, by ANOVA for comparison between combined ST-VPA groups

**statistically significant difference between these two groups, as determined by post hoc Tukey test for comparison between different pairs of means
